# Integrated functional genomic screening to bypass TKI resistance in chronic myeloid leukemia

**DOI:** 10.1016/j.xcrm.2024.101565

**Published:** 2024-05-21

**Authors:** Tumas Beinortas, Brian J.P. Huntly

**Affiliations:** 1 Department of Haematology, University of Cambridge, Cambridge, UK; 2 Wellcome Trust-Medical Research Council Cambridge Stem Cell Institute, Cambridge, UK; 3 Cambridge University Hospitals, Cambridge, UK

## Abstract

CML is readily treatable with tyrosine kinase inhibitors (TKIs); however, resistance occurs, with the disease curable in only ∼15%–20% of patients. Using integrated functional genomics, Adnan Awad et al.^1^ identify agents effective against CML stem cells and describe mechanisms underlying TKI resistance.

## Main text

Tyrosine kinase inhibitors (TKIs) inhibit the disease-defining BCR::ABL1 fusion kinase, are highly effective for chronic myeloid leukemia (CML), and have revolutionized its treatment. With this spectacular success, the optimal therapeutic goal for newly diagnosed CML patients in chronic phase has become achieving sufficiently deep molecular remission for a trial of therapy discontinuation leading to treatment-free remission (TFR). However, only 15%–20% of patients achieve this,^2^ and even later-generation TKI regularly fail to completely eradicate the CML leukemia stem cells (CML-LSCs) that are the reservoir for disease persistence.^3^

Treatment failure, due to either varying degree of primary TKI resistance or acquired secondarily via selective pressure of daily TKI treatment, requires the use of successive advanced-generation ABL1 kinase inhibitors. However, for some patients, adequate disease control is not achieved with currently available BCR::ABL1 inhibitors, because of either continued resistance or toxicity. For fit patients, this leaves stem cell transplantation (SCT) as the only option for long-term disease control/cure, albeit one associated with non-trivial morbidity/mortality risks and requiring an available donor. For patients unsuitable for SCT, no current satisfactory treatment options exist.

Mechanisms of resistance vary from either mutations in the BCR::ABL1 fusion protein itself (genetic resistance) or, more commonly, non-genetic causes related to biological pathways separate from BCR::ABL1 and its signaling cascade. Genetic-resistance mechanisms have been extensively studied, and mutations such as the gatekeeper T315I mutation have propelled the design of more advanced TKIs. In contrast, BCR::ABL1-independent TKI-resistance mechanisms in CML have been barely studied. The only prior study to investigate TKI resistance systematically was limited to a cell-line CRISPR screen under the selective pressure of the first-generation TKI Imatinib.^4^ Consequently, targeted translational efforts for managing TKI-resistant CML remain largely uninformed.

Two major therapeutic questions concern CML patients at opposite ends of the therapeutic spectrum. For TKI responders, how can we achieve reliable and deeper upfront eradication of CML-LSCs, allowing greater TFR and long-term cure? For pan-TKI-resistant patients, what other therapies can be effectively used to achieve improved disease control? Paradoxically, solutions to both paradigms depend on understanding the same biological milieu: molecular drivers of BCR::ABL1-independent CML-LSC fitness. *Ex vivo* drug screening at diagnosis integrated with detailed molecular diagnostics and phenotyping of patients’ disease tissue promises to advance personalization of treatment in hemato-oncology and beyond.^5^^,^^6^^,^^7^ In the current study, Adnan Awad et al. report integration of an *ex vivo* drug screen combined with functional genomics screen to identify (1) the molecular pathways leading to TKI resistance and (2) novel non-TKI agents with specific therapeutic activity against CML-LSCs and thus herald a new era of precision hematology.

To identify effective novel agents against CML-LSCs, the authors performed a comprehensive 82-agent *ex vivo* drug screen on patient-derived CD34^+^ cells—a compartment enriched for CML-LSCs. Comparing the therapeutic index between newly diagnosed CML- patient- and healthy-donor-derived CD34^+^ cells, the authors identify VEGFR, MDM2, WEE1 inhibitors, and Mepacrine as selective inhibitors of CML-derived CD34^+^ cells. Of interest, Mepacrine induced selective differentiation of only the most primitive CD34^+^ CD38^−^cells derived from CML patients, something that even TKIs failed to achieve. The authors next explored the synergy between the most promising single agents and TKIs, hypothesizing that such combinations may achieve more effective eradication of CML-LSCs. While MDM2, BCL2, and WEE1 inhibitors demonstrated synergy with Imatinib, only Navitoclax—a combined BCL2/BCL-X_L_/BCL-w inhibitor—demonstrated ongoing synergy with more potent TKIs. The observed synergy between BCL2 family inhibitors and TKIs in eradicating CML-LSCs corroborates findings from previous studies and highlights a promising clinical strategy in improving medical cure in CML.^8^^,^^9^

To further inform BCR::ABL1-independent TKI-resistance programmes, Adnan Awad et al. performed multiple functional genomics CRISPR-dropout/enrichment screens in CML cell lines under drug treatment-selection pressure, describing shared resistance mechanisms across three generations of TKIs. Of note, the authors identified loss of the ubiquitin ligase substrate adaptor *KCTD5* as a novel TKI resistance mechanism. Moreover, they confirmed KCTD5 as a BCR::ABL1 interactor and demonstrated impaired ubiquitination of BCR::ABL1 upon KCTD5 loss, suggesting that ubiquitination targets BCR::ABL1 for degradation. Interestingly, the VEGFR inhibitor tivozanib was found to share most resistance programs with TKIs, suggesting that it exerts anti-CML therapeutic effects through signaling cascades shared with BCR::ABL1. By contrast, WEE1 inhibitors and Mepacrine did not share resistance programs with TKIs, identifying them to work via parallel pathways and as candidate agents for clinical exploration in pan-TKI-resistant CML.

**Figure 1:**
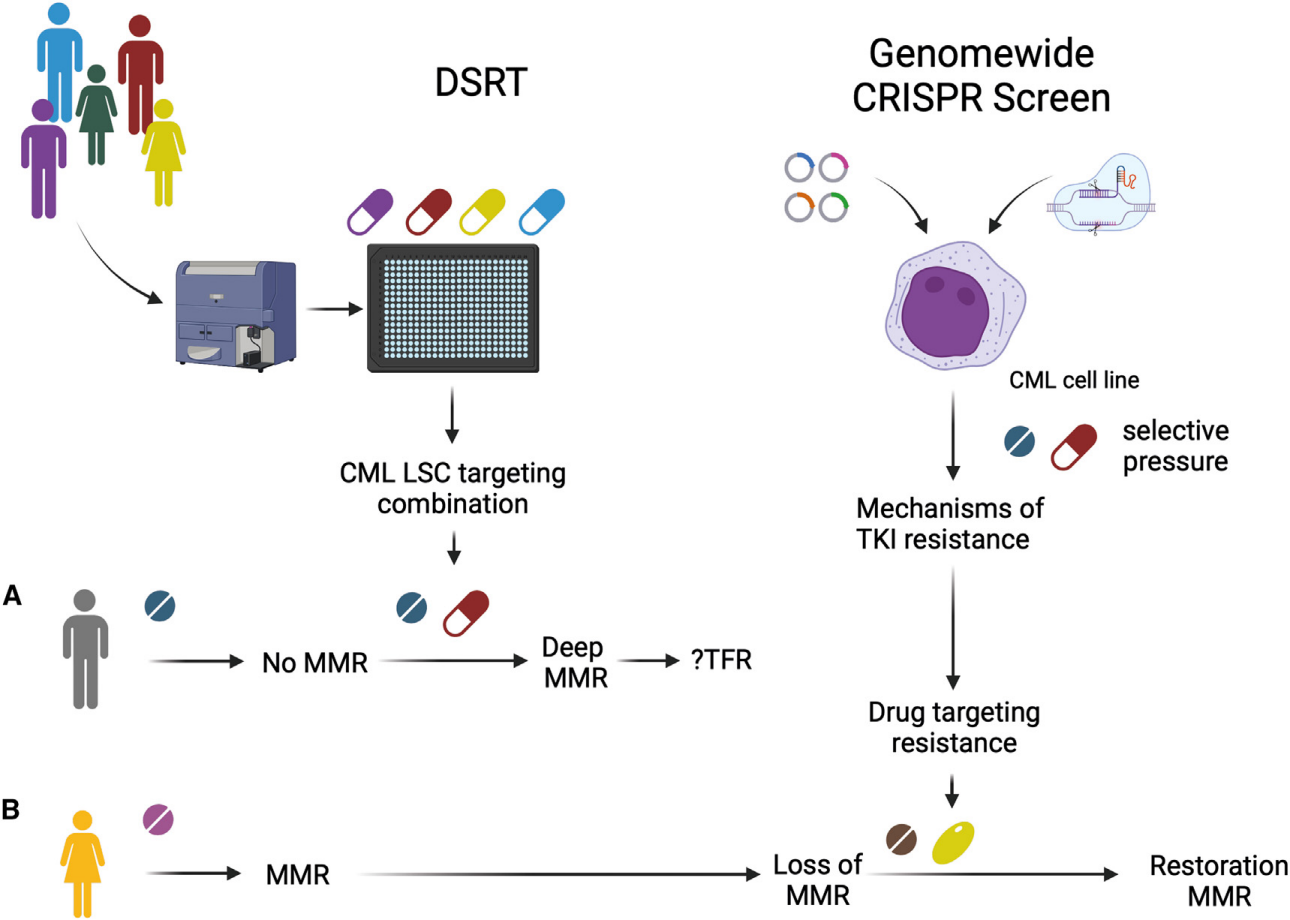
Integrative functional genomics in CML to treat BCR::ABL-independent resistance and increase TFR Adnan Awad and colleagues combine genome-wide CRISPR screening of CML cell lines under selective pressure of TKI therapy with combinatorial drug screening in LSCs from individual CML patients to identify (A) personalized strategies to eradicate LSCs and achieve TFR and (B) novel effective combinations to restore response in resistant patients. DSRT, drug sensitivity and resistance testing; MMR, major molecular response.

The study by Adnan Awad and colleagues is important on several levels (Figure 1). Firstly, performing *ex vivo* drug screening at scale from surface-antigen-selected primary patient samples is technically challenging. Their success further validates *ex vivo* drug screening as a potentially feasible clinical approach in screening LSCs instead of whole-tissue drug susceptibility. Secondly, they also identify multiple novel candidate agents, e.g., MDM2, WEE1 inhibitors, and Mepacrine, as selective agents depleting CML-LSCs through mechanisms separate from TKIs. The utility of these agents warrants further exploration in pan-TKI-resistant CML. Finally, the authors address an important and thus far not molecularly elucidated clinical problem, the molecular basis of BCR::ABL1-independent TKI resistance. The study yields multiple resistance-mediator genes/targets, including *KCTD5*, which require mechanistic understanding and may inform future targeted translational efforts in CML.

Following Adnan Awad and colleagues’ study, further mechanistic studies are required to prove how KCTD5 mediates TKI resistance, how TKI-resistant CML patients downregulate KCTD5, and whether this can be exploited therapeutically. Pharmacological studies will be needed to explore whether the effective concentrations of WEE1, MDM2 inhibitors, and Mepacrine observed in the *ex vivo* studies are clinically achievable with acceptable toxicities, individually and with TKI, before assessing their efficacy in TKI-resistant patients. Finally, performing a similar *ex vivo* drug screen using cells from TKI-resistant patients will be needed to establish *ex vivo* drug screening in CML as an actionable entity in clinical practice, as has been recently shown in acute myeloid leukemia.^5^
